# A single-center retrospective study of factors related to the effects of intravenous glucocorticoid therapy in moderate-to-severe and active thyroid-associated ophthalmopathy

**DOI:** 10.1186/s12902-018-0240-8

**Published:** 2018-02-20

**Authors:** Yang Wang, Shuo Zhang, Yidan Zhang, Xingtong Liu, Hao Gu, Sisi Zhong, Yazhuo Huang, Sijie Fang, Jing Sun, Huifang Zhou, Xianqun Fan

**Affiliations:** 1 Department of Ophthalmology, Ninth People’s Hospital, Shanghai JiaoTong University School of Medicine, No. 639 ZhiZaoJu Road, Shanghai, 200011 China; 2 Shanghai Key Laboratory of Orbital Diseases and Ocular Oncology, Shanghai, China; 3 Shanghai Jiao Tong University School of Medicine, Shanghai, China

**Keywords:** Intravenous glucocorticoid therapy, Thyroid-associated ophthalmopathy, Duration of eye symptoms, Restoration of euthyroidism, Pretreatment clinical activity score

## Abstract

**Background:**

Intravenous glucocorticoids (ivGC) have been recommended as a first-line treatment of moderate-to-severe and active thyroid-associated ophthalmopathy (TAO). However, not all patients are responsive to ivGC. The identification of potential factors used to predict their efficacy and the selection of suitable patients have both been lacking.

**Methods:**

It was a single center retrospective study. Potential factors related to the effects of ivGC were analyzed using logistic regression in 90 consecutive patients with moderate-to-severe and active TAO, who received 4.5 g ivGC therapy. Response was defined as the achievement of at least three points of the overall response.

**Results:**

Fifty-two (57.8%) patients showed a positive response to ivGC therapy. Significant correlations were observed between the effects of ivGC and pretreatment clinical activity score (CAS), duration of eye symptoms, and restoration of euthyroidism. The two latter factors were both independent. The duration of eye symptoms was negatively correlated with the effects of ivGC, with an odds ratio (OR) of 0.984 (*p* = 0.012). Restoration of euthyroidism (OR = 3.282, *p* = 0.039) and pretreatment CAS (OR = 1.653, *p* < 0.01) were both positively correlated with the effects of ivGC. The diagnostic accuracy of the duration of eye symptoms was ≤13 months (*p* = 0.000), with a specificity of 76.9%, and sensitivity of 65.8%. The diagnostic accuracy of the pretreatment CAS was more than 2.5 (*p* = 0.000), with a specificity of 61.5% and sensitivity of 80.5%. Besides, a multi-variables prediction model were established as well, which was better in the forecasting aspect with an area under curve of 0.784 (*p* = 0.000).

**Conclusions:**

The duration of eye symptoms and restoration of euthyroidism are independent factors that are associated with the effects of ivGC. The following practical implications were inferred: firstly, the shorter the duration of eye symptoms, the more favorable the effects of ivGC therapy. Thus, prompt diagnosis and treatment (within 13 months) is important. Secondly, the restoration of euthyroidism improves the efficacy of ivGC. Thirdly, hope the multi-variables prediction model can be applied to clinical therapy in the future.

## Background

Thyroid-associated ophthalmopathy (TAO) is an autoimmune inflammatory disorder that can lead to permanent vision loss. Common clinical manifestations include eyelid retraction (90%), exophthalmos (60%), and restricted extraocular motility (40%) requiring medical intervention [1]. The various interventions can differ according to the characteristics of the patients and stages of the disease, and include the restoration of euthyroidism in all patients; selenium supplementation for those affected by mild TAO; immunosuppressive therapy and radiotherapy for active TAO; and decompression surgery for emergency or inactive TAO [2]. For active TAO, glucocorticoids continue to be the primary immunosuppressive therapy, and some steroid-sparing regimens include rituximab and teprotumumab, whereas other regimens also prove to be effective [3]. Recent research has provided insight into potential differences in the effects of glucocorticoid therapy, based on various routes of administration (oral, intravenous, or local injection) [4–6]; doses; and schedules (weekly or daily) [7]. In the recent consensus statement of the European Group on Graves’ Orbitopathy (EUGOGO), high-dose intravenous glucocorticoid (ivGC) therapy is recommended as the first-line treatment for moderate-to-severe and active TAO, and immediate therapy for sight-threatening TAO [2]. The response rate of ivGC appears to be approximately 70%–80% according to several randomized clinical trials [7, 8], suggesting that the remaining 20%–30% of all patients show no response and require additional therapy. Unfortunately, some of these patients have possibly missed their best treatment opportunities. Ineffective therapy can be a waste of resources. Thus, it is necessary to evaluate the prognoses and selection of suitable patients for ivGC. This study was designed to identify some of the potential factors associated with the effects of ivGC that can be used to predict their efficacy and facilitate the selection of suitable patients.

## Methods

This retrospective study was approved by the Ethics Committee of Shanghai Ninth People’s Hospital. It was in compliance with the tenets of the Declaration of Helsinki for clinical research.

### Study design and patients

This study retrospectively reviewed a cohort of all consecutive patients diagnosed with active TAO, who had received 4.5 g intravenous methylprednisolone therapy. Patients were divided into two groups, based on their response to ivGC. The evaluation of response was based on the “overall response [5, 7].” Logistic regression models were then used to predict potential factors that were associated with the effects of ivGC therapy.

A total of 90 patients were included in the study, who presented to the Department of Ophthalmology, Shanghai Ninth People’s Hospital, Affiliated Shanghai Jiao Tong University School of Medicine from December 2014 to December 2016. Diagnosis of TAO was based on the EUGOGO consensus [2]. Active TAO was defined by a.) clinical activity score (CAS) ≥3/7; or b.) CAS < 3/7, but with increased signal intensity of extraocular muscles on contrast-enhanced orbital magnetic resonance imaging (MRI). The CAS was also based on the EUGOGO consensus [2]. The protocol was as follows: 0.5 g per week for 6 weeks, followed by 0.25 g per week for 6 weeks. During the course of treatment, proton pump inhibitors or H2-receptor antagonists and calcium supplements were prescribed for every patient. Anti-thyroid drugs were used to restore and maintain euthyroidism. The detailed inclusion and exclusion criteria are presented in Table 1 and Table 2.

**Table 1:** Inclusion criteria

1 Diagnosed with TAO	
2 CAS ≥3/7	
3 Contrast-enhanced orbital MRI showed increased signal intensity of extraocular muscles	
4 Received 4.5 g intravenous methylprednisolone therapy	

Patients should have met either criteria 2 or 3, together with criteria 1 and 4 to be included in the study. *TAO* thyroid-associated ophthalmopathy, *CAS* clinical activity score, *MRI* magnetic resonance imaging

**Table 2:** Exclusion criteria

1 Received orbital radiotherapy, decompression surgery, or other immunosuppressive therapy within the previous 3 months or during ivGC therapy.	
2 Abnormal heart, liver and kidney function.	

ivGC, intravenous glucocorticoid

### Clinical assessment and response evaluation

Demographic characteristics were compiled before treatment. The mean follow-up time was four weeks. Thyroid function and ophthalmic assessment that included the incidence of proptosis, eyelid width, diplopia, intraocular pressure, visual acuity, and CAS were compared before and after treatment (4 weeks after 4.5 g ivGC treatment). The classifications of diplopia were as follows: Grade 0, no diplopia; Grade 1, diplopia with horizontal or vertical gaze; Grade 2, intermittent diplopia in straight gaze; and Grade 3, constant diplopia in straight gaze. Eyelid width was recorded as the margin reflex distance (MRD), that is, the distance between the pupil center and eyelid margin. The MRD-1 was the distance between the pupil center and upper eyelid margin, whereas MRD-2 was the distance between the pupil center and lower eyelid margin.

Patients were divided into two groups, “responsive” and “unresponsive.” The “responsive” group was defined as those manifesting at least three of the outcome measures (Table 3). Gender, age, duration of eye symptoms, smoking history, family history of TAO, history of impaired thyroid function, pretreatment CAS, and restoration of euthyroidism were analyzed to identify potential factors associated with the response to ivGC therapy. Gender was scored as male 1 and female 0. Smoking history, family history of TAO, and history of impaired thyroid function were scored as yes 1 or no 0. If thyroid function was impaired before treatment and restored after treatment, we scored the restoration of euthyroidism as 1. If thyroid function was still abnormal or was not impaired at the beginning, a score of 0 was recorded.

**Table 3:** Overall response

1 Reduction in lid width by at least 3 mm	
2 Reduction in any of the class 2 NO SPECS signs by at least two grades	
3 Reduction in intraocular pressure by at least 2 mmHg	
4 Reduction in proptosis by at least 2 mm	
5 Improvement in CAS by at least two points	
6 Improvement in diplopia (disappearance or lessening of the degree)	
7 Improvement in visual acuity by 1 Snellen score	

Determination of overall response was based on several studies [5, 7]; NO SPECS was based on the EUGOGO consensus [2]; *CAS* clinical activity score

### Statistical analysis

Continuous variables were reported as the median ± SD or median [interquartile intervals, 25th–75th percentile], and categorical variables were reported as percentages. Continuous variables were compared using the Mann–Whitney U-test. Categorical variables were compared using the χ [2] test or Mann–Whitney U-test, as appropriate. Simple logistic regression was used to identify potential parameters associated with the effects of ivGC therapy. Multivariable logistic regression was then used to determine statistically significant outcomes. Diagnostic accuracy was evaluated using receiver operating characteristic (ROC) curve analysis. Statistical significance was defined as *p* < 0.05. All statistical analyses were performed by two authors and a statistician, using the SPSS 13.0 software (SPSS, Chicago, IL, USA).

## Results

Ninety patients (32 males, 58 females; mean age ± SD, 44.61 ± 15.06 years; age range 15–85 years) were included in the study. All patients were Asians. Thirty-three (36.7%) patients presented with a pretreatment CAS < 3/7, but had increased signal intensity of extraocular muscles on contrast-enhanced orbital MRI. Fifty-two (57.8%) patients who showed a response to ivGC therapy were defined as “responsive,” and the other 38 (42.2%) were defined as “unresponsive”. The mean duration of eye symptoms was 8.75 months (range of 4–12 months) in the responsive group, and 24 months (12–75 months) in the unresponsive group, with an OR of 0.984 (95% confidence interval [CI], 0.971–0.996, *p* = 0.011). The mean pretreatment CAS in the two groups were 4 (3, 4) (responsive) and 2 (1–3) (unresponsive), with an OR of 1.653 (95% CI, 1.214–2.250, *p* = 0.001). The OR of the restoration of euthyroidism was 3.272 (95% CI, 1.150–9.315, *p* = 0.026). Generally, these three factors were identified as parameters that were associated with the effects of ivGC therapy (Table 4). However, gender, age, smoking history, family history of TAO, and a history of impaired thyroid function were not identified as parameters that were associated with the effects of ivGC therapy.

**Table 4:** Results of simple logistic regression

Factor	Responsive	Unresponsive	*p*-Value	OR	95% CI of OR	
					Lower	Upper
Gender, males	21 (40.4%)	11 (28.9%)	0.265	1.663	0.680	4.063
Age, years	46.75 ± 12.95	41.68 ± 17.3	0.117	1.023	0.994	1.053
Duration of eye symptoms, months	8.75 [4–12]^a^	24 [12–75]	0.011	0.984	0.971	0.996
Smoking history	28 (53.8%)	19 (50%)	0.718	1.167	0.505	2.696
Family history of TAO	9 (17.3%)	12 (31.6%)	0.118	0.453	0.168	1.223
History of impaired thyroid function	44 (84.6%)	36 (94.7%)	0.149	0.306	0.061	1.530
Pretreatment CAS	4 [3–4]	2 [1–3]	0.001	1.653	1.214	2.250
Restoration of euthyroidism	19 (36.5%)	6 (15.8%)	0.026	3.272	1.150	9.315

*OR* Odds ratio, *CI* confidence interval, *TAO* thyroid-associated ophthalmopathy, *CAS* clinical activity score, ^a^[interquartile intervals, 25th–75th percentile]

We analyzed the interactions among the three variables. Significant interactions were observed between the pretreatment CAS and both the duration of eye symptoms and restoration of euthyroidism (Table 5). After excluding the pretreatment CAS from our analysis, multivariable logistic regression was performed for the other two variables: the duration of eye symptoms and restoration of euthyroidism.

**Table 5:** Results of interaction analysis

Factor	*p*-value	OR	95.0% CI of OR	
			Lower	Upper
Pretreatment CAS by duration of eye symptoms	0.002	0.983	0.972	0.994
Pretreatment CAS by restoration of euthyroidism	0.020	1.453	1.059	1.992
Duration of eye symptoms by restoration of euthyroidism	0.215	0.987	0.966	1.008

*OR* Odds ratio, *CI* confidence interval, *CAS* clinical activity score

Both variables were found to be associated with the effects of ivGC therapy (Table 6). The OR of the duration of eye symptoms was 0.984 (95% CI, 0.972–0.997, *p* = 0.012), and that of the restoration of euthyroidism was 3.282 (95% CI, 1.062–10.142, *p* = 0.039). Therefore, a shorter duration of eye symptoms led to greater efficacy of the therapy. More specifically, the earlier therapy commenced, the more favorable were the effects observed.

**Table 6:** Results of multivariable logistic regression

Factor	*p*-value	OR	95.0% CI of OR	
			Lower	Upper
Duration of eye symptoms	0.012	0.984	0.972	0.997
Restoration of euthyroidism	0.039	3.282	1.062	10.142

*OR* Odds ratio, *CI* confidence interval

**Fig. 1:**
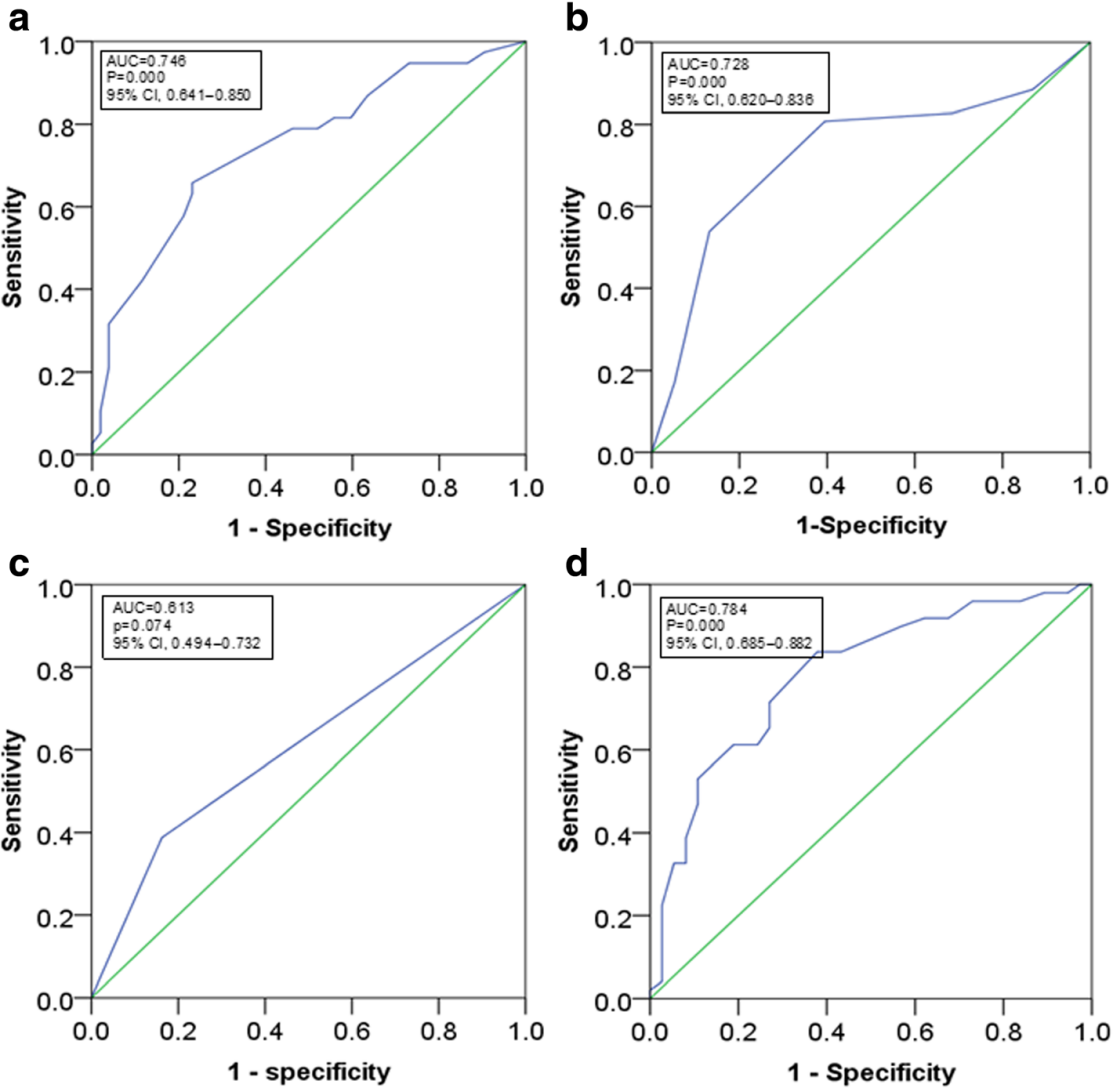
Receiver operating characteristic (ROC) curve analysis. **a** duration of eye symptoms; **b** pretreatment CAS; **c** restoration of euthyroidism (a binary categorical variable); **d** a multivariable prediction model with variables of the duration of eye symptoms and restoration of euthyroidism. *ROC* receiver operating characteristic, *AUC* area under curve, *CI* confidence interval, *CAS* clinical activity score

The diagnostic accuracy of the duration of eye symptoms was 13 months (95% CI, 0.641–0.850, *p* = 0.000), based on ROC curve analysis, with an area under curve (AUC) of 0.746, specificity of 76.9% and sensitivity of 65.8% (Fig. 1a). The diagnostic accuracy of the pretreatment CAS was 2.5 (95% CI, 0.620–0.836, *p* = 0.000), with an AUC of 0.728, specificity of 61.5% and sensitivity of 80.5% (Fig. 1b). The ROC curve analysis of restoration of euthyroidism showed an AUC of 0.613 (95% CI, 0.494–0.732, *p* = 0.074) (Fig. 1c). Furthermore, a multivariable prediction model was established with variables of the duration of eye symptoms and restoration of euthyroidism. The ROC curve analysis showed an AUC of 0.784 (95% CI, 0.685–0.882, *p* = 0.000) (Fig. 1d), which was better than those single-variable prediction models.

## Discussion

Our study found three potential factors that were associated with the effects of ivGC therapy. A higher CAS indicated a stronger inflammatory reaction and increased disease activity that was likely to be alleviated following ivGC therapy [9]. Interaction analysis revealed significant interactions between the pretreatment CAS and duration of eye symptoms. These two parameters were not independent of each other. These findings are consistent with those from clinical experience: patients were more likely to have inactive TAO with a longer duration of eye symptoms, whereas inactive TAO was characterized by a lower CAS. The existing cut-off point between active TAO and inactive TAO was a CAS of 3. A CAS ≥3/7 was indicative of active TAO [9–11]. The ivGC therapy administered as a form of immunosuppressive therapy proved to be effective against active TAO, and based on the pathogenesis of TAO, was also an acceptable choice [7, 8]. The recent consensus statement of EUGOGO recommended high-dose ivGC as the first-line treatment for patients with a CAS ≥3/7 [2]. However, the inclusion criteria of our study were not restricted to a pretreatment CAS ≥3/7. Some patients with a CAS < 3/7 exhibited inflammatory signs, such as soft tissue swelling or enlargement of the extraocular muscles on contrast-enhanced MRI. We found glucocorticoids to be generally effective in treating these symptoms. Thus, we decided to include patients with a CAS < 3/7 who exhibited an increased signal intensity of the extraocular muscles on contrast-enhanced MRI in our analysis as well.

Based on the results of ROC analysis, we found that ivGC therapy was more effective when the CAS was > 2.5. However, these results might have been limited by the relatively small sample size. Furthermore, the criteria established in previous studies [9, 10] were not designed for Asian patients. We believe that the cut-off point for the CAS of Asian patients might be < 3. This is because of the differences in orbital anatomy between Caucasians and Asians; Asian eyelids tend to have more subcutaneous and suborbicularis fat [12]. The thicker eyelid might lead to reduced orbital inflammation. In one Chinese study [7], only 5.13%–10.26% of Asian patients presented with eyelid redness, whereas a Dutch study [10] reported this figure to be less than 50%. Nevertheless, this theory would require further investigation to be confirmed. Randomized controlled trials on larger sample sizes, specifically designed for Chinese patients are recommended for further research.

Interactions were also noted between the pretreatment CAS and restoration of euthyroidism. The reason for this interaction remains unclear, based on our present clinical knowledge. After the parameter of the pretreatment CAS was excluded, and multivariable logistic regression for the other two variables was conducted, we found that both the duration of eye symptoms and restoration of euthyroidism could have affected the effects of ivGC therapy. Patients with a shorter duration of eye symptoms were more likely to be at an active phase of the condition, and were thus more likely to respond to glucocorticoid therapy. We also found that ivGC therapy was more likely to be effective when patients were administered the therapy within 13 months after the first appearance of eye symptoms. Therefore, prompt diagnosis and treatment are recommended.

The restoration of euthyroidism indicated an impairment of thyroid function before treatment and a subsequent restoration before the last follow-up. Thus, a patient with impaired thyroid function became euthyroid following therapy, and was generally more responsive to ivGC therapy. These findings could be attributed to the concurrent administration of anti-thyroid drugs. Methimazole is a common drug indicated for thyroid dysfunction. According to previous studies, it is also an immunosuppressant [13, 14]. Thus, we speculated that some anti-thyroid drugs could alleviate inflammation of the orbital tissue and improve the overall efficacy of glucocorticoids. Prompt restoration and maintenance of euthyroidism is recommended for TAO patients, both to enhance thyroid function and improve the efficacy of ivGC therapy.

Besides, a multivariable prediction model was established including variables of the duration of eye symptoms and restoration of euthyroidism, which was better in the forecasting aspect than those single-variable prediction models with an AUC of 0.784. We hope to verify this model in larger cohorts. Hope it can be used to predict ivGC efficacy and facilitate the selection of suitable patients.

One study conducted in Italy reported that age and glucocorticoid dose were major risk factors for liver damage, a common side effect of ivGC therapy in TAO [15]. Researchers have also reported that the response to ivGC therapy increases with time [16]. Biomarkers of serum microRNA-224-5p and thyroid-stimulating hormone receptor antibody (TRAb) have been observed to predict glucocorticoid sensitivity [17]. In another study [18], smoking is reportedly associated with a poor response to glucocorticoid therapy. However, this finding is not consistent with the present results. One possible reason might be the different grouping methods. In that study, they divided the patients into three groups: Never-smokers (23 patients), active smokers (31 patients), and passive smokers (38 patients). Patients who had never smoked throughout their lives and had never breathed in the smoke from other people’s cigarettes throughout their lives were considered as never smokers. Patients who had never smoked themselves but breathed in the smoke from other people’s cigarettes were considered as passive smokers. The others were considered as active smokers. They compared active smokers and never smokers. They found that never-smokers showed a significantly higher response rate than active smokers [18]. In our study, we divided patients in two groups: group 1 included active smokers and group 2 included never smokers and passive smokers. We compared the two groups and inferred that smoking was not a factor that was significantly associated with the effects of ivGC therapy. Another study also reported that smoking was not a factor that affects the efficacy of ivGC therapy [16]. They divided patients in the same way as ours. Thus, we inferred that passive smokers might have interfered with the analysis. Further research should be conducted to determine the nature of this interference. Another limitation of our study was the follow-up time that was not long enough to determine the long-term effects of glucocorticoid administration.

## Conclusion

In patients with TAO, a shorter duration of eye symptoms (≤13 months) and the restoration of euthyroidism indicated greater efficacy of ivGC therapy. The pretreatment CAS was not an independent factor, but was an important parameter for the prediction of efficacy and selection of suitable patients. A multivariable prediction model had been established. We hope it can be applied to clinical therapy in the future. Based on the present results and other recent studies, active smoking history could also be a potential factor that can affect the efficacy of ivGC therapy.

## Abbreviations

AUC: area under curve
CAS: clinical activity score
CI: confidence interval
EUGOGO: European Group on Graves’ Orbitopathy
ivGC: intravenous glucocorticoid
MRD: margin reflex distance
MRI: magnetic resonance imaging
OR: odds ratio
ROC: receiver operating characteristic
TAO: thyroid-associated ophthalmopathy
TRAb: thyroid-stimulating hormone receptor antibody
